# Cross-cultural adaptation and psychometric validation of the Chinese version of the empathy fatigue scale for health professionals among intensive care unit nurses

**DOI:** 10.3389/fpsyt.2026.1918940

**Published:** 2026-07-29

**Authors:** Zhuona Zhang, Peiwei Zou, Xinchen Yang, Min Yang, Wei wei Wang, Huimin Li, Xin Guan, Xuelian Xu

**Affiliations:** 1 School of Nursing, Inner Mongolia Medical University, Hohhot, Inner Mongolia, China; 2 Nanchang Institute of Technology, Nanchang, Jiangxi, China; 3 School of Economics and Management, Jiangxi Normal University, Nanchang, Jiangxi, China; 4 Tongliao People's Hospital, Tongliao, Inner Mongolia, China; 5 School of Medicine, Nanchang Institute of Technology, Nanchang, Jiangxi, China; 6 Inner Mongolia Autonomous Region People's Hospital, Hohhot, Inner Mongolia, China; 7 Peking University Cancer Hospital Inner Mongolia Hospital, Hohhot, Inner Mongolia, China

**Keywords:** cross-cultural adaptation, empathy fatigue, ICU nurses, intensive care unit, mental health, psychometric validation

## Abstract

**Introduction:**

Intensive care unit (ICU) nurses are chronically exposed to patient suffering, death, and morally complex decisions, placing them at elevated risk of empathy fatigue. China currently lacks a psychometrically sound instrument developed specifically to capture empathy fatigue within the ICU specialty context.

**Objective:**

This study aimed to cross-culturally adapt the Empathy Fatigue Scale for Health Professionals (EFS-HP) into Chinese and evaluate its psychometric properties among ICU nurses.

**Methods:**

The EFS-HP was translated and adapted following Sousa’s seven-step framework and Brislin’s translation model. A convenience sample of 300 ICU nurses from a tertiary grade-A hospital in the Inner Mongolia Autonomous Region completed the Chinese EFS-HP and the Compassion Fatigue Scale for Medical Staff (CFS-MS). Item analysis, reliability testing (internal consistency, split-half, test–retest), and validity testing (content validity, exploratory and confirmatory factor analyses, convergent validity) were performed using SPSS 27.0 and AMOS 31.0.

**Results:**

The Chinese EFS-HP retained all 21 items but yielded a two-factor structure—Emotional Load and Exhaustion State—accounting for 78.165% of the total variance. The scale demonstrated excellent internal consistency (Cronbach’s α = 0.981), split-half reliability (Spearman–Brown = 0.938), and test–retest reliability (r = 0.958). Confirmatory factor analysis supported the two-factor model over the original single-factor model. The total score showed a moderate and statistically significant positive correlation with the CFS-MS (r = 0.587, P < 0.001), supporting convergent validity.

**Conclusion:**

The Chinese EFS-HP is a reliable and valid instrument for assessing empathy fatigue among ICU nurses and may serve as a practical screening tool to inform targeted mental health interventions.

## 1 Introduction

Empathy is widely regarded as foundational to the therapeutic nurse–patient relationship and to the delivery of high-quality, person-centered care (1). Yet the very capacity that enables nurses to connect with suffering patients also exposes them to a distinct occupational hazard (2). Intensive care unit (ICU) nurses, in particular, are chronically immersed in environments characterized by acute patient suffering, traumatic events, frequent mortality, and morally complex decision-making (3, 4). This sustained and intense emotional investment can progressively erode a clinician’s empathic resources—a phenomenon increasingly conceptualized as empathy fatigue (5–7).

Empathy fatigue carries consequences that extend well beyond the individual clinician. At the personal level, it is associated with depression, anxiety, and sleep disturbance (8). At the professional level, it diminishes job satisfaction and heightens turnover intention (9). Ultimately, the depletion of empathic capacity may compromise the quality and safety of patient care, making empathy fatigue a pressing concern for both mental health and health-system sustainability (9, 10).

Empathy fatigue is conceptually related to, but distinguishable from, other established occupational constructs such as burnout, compassion fatigue, and secondary traumatic stress. Burnout is generally understood as a syndrome of emotional exhaustion, depersonalization, and reduced personal accomplishment resulting from chronic occupational stress (39, 40). Secondary traumatic stress refers to trauma-related symptoms that arise from indirect exposure to others’ traumatic experiences (29). Compassion fatigue has been described as a broader construct that may reflect the combined effects of burnout and secondary traumatic stress, although its conceptual boundaries remain subject to ongoing discussion (29, 41).In contrast, empathy fatigue is characterized by a gradual depletion of empathic capacity resulting from sustained emotional engagement with patients’ suffering (19). Clarifying these conceptual distinctions is important for ensuring the appropriate selection and cross-cultural adaptation of measurement instruments in critical care settings.

Despite growing recognition of this construct, China currently lacks a measurement instrument developed specifically for the ICU specialty setting. Existing general-purpose scales, while useful, often fail to capture the particular forms of emotional exhaustion and empathic depletion that characterize critical care nursing (11, 12). Addressing this gap, the present study sought to cross-culturally adapt the Empathy Fatigue Scale for Health Professionals (EFS-HP), originally developed by Okan et al., into Chinese, and to rigorously evaluate its reliability and validity among Chinese ICU nurses (13, 14). In doing so, we aimed to provide a culturally appropriate, psychometrically sound tool to support the early identification of at-risk nurses and the design of localized mental health interventions.

## 2 Methods

### 2.1 Study design

This was a methodological, cross-sectional psychometric study.

### 2.2 Cross-cultural adaptation procedure

The adaptation process followed Sousa’s seven-step approach and Brislin’s translation model (15):

Forward translation: Two bilingual nursing experts independently translated the original EFS-HP into Chinese (T1: master’s degree; T2: doctoral degree).

Synthesis: The research team reconciled discrepancies based on semantic and conceptual equivalence and Chinese nursing language conventions.

Back-translation: Two bilingual translators blinded to the original instrument independently back-translated the synthesized Chinese version (T3: doctoral degree, IELTS background; T4: master’s degree, CET-6).The specific information of the translation team is detailed in **Table 1**.

**Table 1:** Profiles of the EFS-HP scale translation team.

No	Educational background	English proficiency	Occupation	Task	Self-assessment of familiarity with the scale
T1	Master’s	CET-6	Co-chief Nurse	Translation	Moderately familiar
T2	Doctorate	CET-6	Nurse	Translation	Highly familiar
T3	Doctorate	IELTS	Teacher	Back-translation	Not familiar
T4	Master’s	CET-6	Teacher	Back-translation	Not familiar

Expert committee review: Six experts in psychology, education, ICU clinical nursing, and methodology assessed semantic clarity, conceptual consistency, and cultural relevance (16).

Pilot testing: Thirty ICU nurses completed the prefinal version to assess comprehensibility and acceptability; no item deletion was required.

### 2.3 Participants and setting

Participants were recruited by convenience sampling from the ICU of a tertiary Grade A hospital in Inner Mongolia Autonomous Region, China, between February and March 2026.

Inclusion criteria.

- Registered nurse licensure.
- At least one year of ICU work experience.
- Voluntary participation.

Exclusion criteria:

- On leave during the data collection period (e.g., maternity leave, sick leave).
- Refusal to participate.

A total of 318 questionnaires were distributed. To ensure data quality, all questionnaires were administered anonymously in a private, independent setting, and participants were clearly informed of the study purpose prior to completion. Only questionnaires completed within 5 to 10 minutes were retained as valid; those with completion times shorter than 5 minutes or longer than 10 minutes were excluded to minimize careless or interrupted responses. After this screening, 300 valid questionnaires were returned, yielding an effective response rate of 94.3%. The final sample size met the commonly recommended criterion of 5–10 participants per item, thereby supporting the planned exploratory and confirmatory factor analyses (17, 18).

### 2.4 Instruments

Demographic questionnaire (self-developed): Collected information on age, sex, marital status, professional title, years of work, perceived stress, and previous empathy fatigue experience.

Chinese version of the EFS-HP: The adapted scale comprised 21 items rated on a 5-point Likert scale (1 = strongly disagree, 5 = strongly agree), yielding total scores ranging from 21 to 105, with higher scores indicating greater empathy fatigue (19).

The Compassion Fatigue Scale for Medical Staff (CFS-MS) (20), developed by Li Xiaoqin (2011) in her master’s thesis at Southwest University, is a 36-item self-report instrument designed to assess compassion fatigue among healthcare professionals in China. Exploratory factor analysis revealed a six-factor structure—mental tension, loss of passion, negative behavior, apathy, doubt of ability, and loss of fighting will—which collectively explain 50.224% of the total variance. Each item is rated on a 5-point Likert scale (1 = strongly disagree to 5 = strongly agree), with total scores ranging from 36 to 180; higher scores indicate more severe compassion fatigue. Psychometric evaluation demonstrated satisfactory internal consistency, with a Cronbach’s α of 0.879 for the total scale and subscale α values ranging from 0.701 to 0.818. Confirmatory factor analysis further supported the model fit (RMSEA = 0.060).

The CFS-MS was selected as the reference measure for convergent validity for several reasons. First, it shares a common theoretical foundation with the EFS-HP, both being grounded in Figley’s Secondary Traumatic Stress Theory and the burnout model. Second, its target population—hospital-based medical staff—is consistent with that of the EFS-HP. Third, its multidimensional structure allows for a comprehensive examination of the unidimensional EFS-HP, making it an appropriate criterion for validating the latter.

### 2.5 Statistical analysis

Data were analyzed using SPSS 27.0 and AMOS 31.0. Item analysis: Critical ratio (CR) and corrected item–total correlation. Reliability: Cronbach’s α coefficient, split-half reliability (Spearman–Brown and Guttman coefficients), and two-week test–retest reliability (21–23). Content validity: Item-level content validity index (I-CVI) and scale-level average content validity index (S-CVI/Ave) (16). Construct validity: Exploratory factor analysis (EFA) and confirmatory factor analysis (CFA) (24, 25). Convergent validity: Pearson correlation between the total scores of the EFS-HP and the CFS-MS (26). A two-sided P value < 0.05 was considered statistically significant. Model fit for CFA was evaluated using the following indices and thresholds: χ²/df < 3, RMSEA < 0.08, CFI > 0.90, TLI > 0.90, IFI > 0.90, and NFI > 0.90 (25).

### 2.6 Ethical considerations

This study was conducted in accordance with the Declaration of Helsinki and approved by the Ethics Committee of the Inner Mongolia Autonomous Region People’s Hospital. All participants provided written informed consent before completing the questionnaires. Anonymity and confidentiality were strictly maintained throughout the study.

## 3 Results

### 3.1 Demographic characteristics

The demographic and occupational characteristics of the 300 participants are summarized in **Table 2**. The sample was predominantly female (87.0%), with most respondents aged 31–40 years (65.33%) and holding more than 10 years of work experience (67.33%). Notably, 80.67% reported high perceived stress over the preceding six months, and 56.67% reported having previously experienced empathy fatigue. .

**Table 2:** Demographic and occupational characteristics of participants (N = 300).

Variable	Category	n	%
Sex	Male	39	13.00
	Female	261	87.00
Age(years)	21-30	57	19.00
	31-40	196	65.33
	≥41	47	15.67
Marital status	Unmarried	58	19.33
	Married	239	79.67
	Divorced	3	1.00
Have children	No	85	28.33
	Yes	215	71.67
Educational background	Master’s degree or above	2	0.67
	Bachelor’s degree	289	96.33
	Associate degree	9	3.00
Years of work experience	1–3 years	18	6.00
	3–5 years	26	8.67
	5–10 years	54	18.00
	≥10years	202	67.33
Professional title	Junior	83	27.67
	Intermediate	146	48.67
	Associate senior	62	20.67
	Senior	9	3.00
Position	Chief nurse	4	1.00
	Head nurse	50	16.00
	Nurse	246	82.00
Perceived high stress in the past six months	Yes	242	80.67
	No	58	19.33
Regular physical exercise	Never	75	25.00
	Rarely	189	63.00
	Often	36	12.00
Previous experience of empathy fatigue	Yes	170	56.67
	No	130	43.33

### 3.2 Item analysis

All 21 items demonstrated strong discriminative capacity. Critical ratio values ranged from 12.627 to 32.826 (*P* < 0.001), and corrected item–total correlations ranged from 0.644 to 0.893, exceeding the conventional threshold of 0.40. Accordingly, all items were retained. Specific values are shown in **Table 3**.

**Table 3:** Item analysis of Chinese EFS-HP.

Item	CR	P	Corrected item-total correlation	Cronbach’s α if item deleted
Q1	12.627	<0.001	0.644	0.981
Q2	15.676	<0.001	0.742	0.981
Q3	21.097	<0.001	0.831	0.980
Q4	23.289	<0.001	0.833	0.980
Q5	23.727	<0.001	0.844	0.980
Q6	24.673	<0.001	0.825	0.980
Q7	24.162	<0.001	0.860	0.980
Q8	26.515	<0.001	0.827	0.980
Q9	32.826	<0.001	0.893	0.979
Q10	26.075	<0.001	0.872	0.979
Q11	25.183	<0.001	0.888	0.979
Q12	18.907	<0.001	0.808	0.980
Q13	21.366	<0.001	0.842	0.980
Q14	22.028	<0.001	0.877	0.979
Q15	21.751	<0.001	0.851	0.980
Q16	20.196	<0.001	0.845	0.980
Q17	18.512	<0.001	0.839	0.980
Q18	20.172	<0.001	0.846	0.980
Q19	23.666	<0.001	0.880	0.979
Q20	19.739	<0.001	0.844	0.980
Q21	17.293	<0.001	0.815	0.980

### 3.3 Reliability

The Chinese EFS-HP exhibited excellent reliability across all indices. Internal consistency was high, with an overall Cronbach’s α of 0.981. Split-half reliability yielded a Spearman–Brown coefficient of 0.938 and a Guttman coefficient of 0.935. Test–retest reliability was assessed over a two-week interval in a subsample of participants (n = 30). Pearson correlation analysis showed excellent temporal stability between the two administrations of the total score (r = 0.958, 95% CI [0.913, 0.980], P < 0.001).

### 3.4 Validity

#### 3.4.1 Content validity

Expert evaluation produced item-level content validity indices (I-CVI) ranging from 0.83 to 1.00 and a scale-level content validity index (S-CVI/Ave) of 0.88, reflecting strong expert consensus on the relevance of items to the target construct.

#### 3.4.2 Construct validity

##### 3.4.2.1 Exploratory factor analysis

Exploratory factor analysis was conducted using principal component analysis (PCA) with varimax orthogonal rotation. The Kaiser–Meyer–Olkin measure indicated excellent sampling adequacy (KMO = 0.969), and Bartlett’s test of sphericity was significant (P < 0.001), confirming suitability for factor analysis. No missing data were observed in the dataset.

Factors were retained based on eigenvalues greater than 1 and inspection of the scree plot. Two factors were extracted, accounting for 78.165% of the total variance.

The first factor had an eigenvalue of 15.230 and accounted for 72.526% of the total variance, while the second factor had an eigenvalue of 1.184 and accounted for 5.639%.

- Factor 1 — Emotional Load (Q9, Q11–Q21; 12 items), reflecting emotional intrusion and psychological burden.
- Factor 2 — Exhaustion State (Q1–Q8, Q10; 9 items), reflecting depletion of physical and psychological resources.

##### 3.4.2.2 Confirmatory factor analysis

Confirmatory factor analysis was conducted to compare a single-factor model with a two-factor structure identified in the exploratory factor analysis (EFA). The model was estimated using maximum likelihood estimation (ML).

The two-factor model demonstrated better fit than the single-factor model. Fit indices for the two-factor model were: χ²/df = 4.361, CFI = 0.923, IFI = 0.923, TLI = 0.911, NFI = 0.902, and RMSEA = 0.106.

Although the RMSEA slightly exceeded the conventional cutoff of 0.08, the incremental fit indices (CFI, IFI, TLI, and NFI) were close to or above 0.90, and the two-factor model consistently showed superior fit compared with the single-factor model, supporting the factorial validity of the scale.

**Figure 1:**
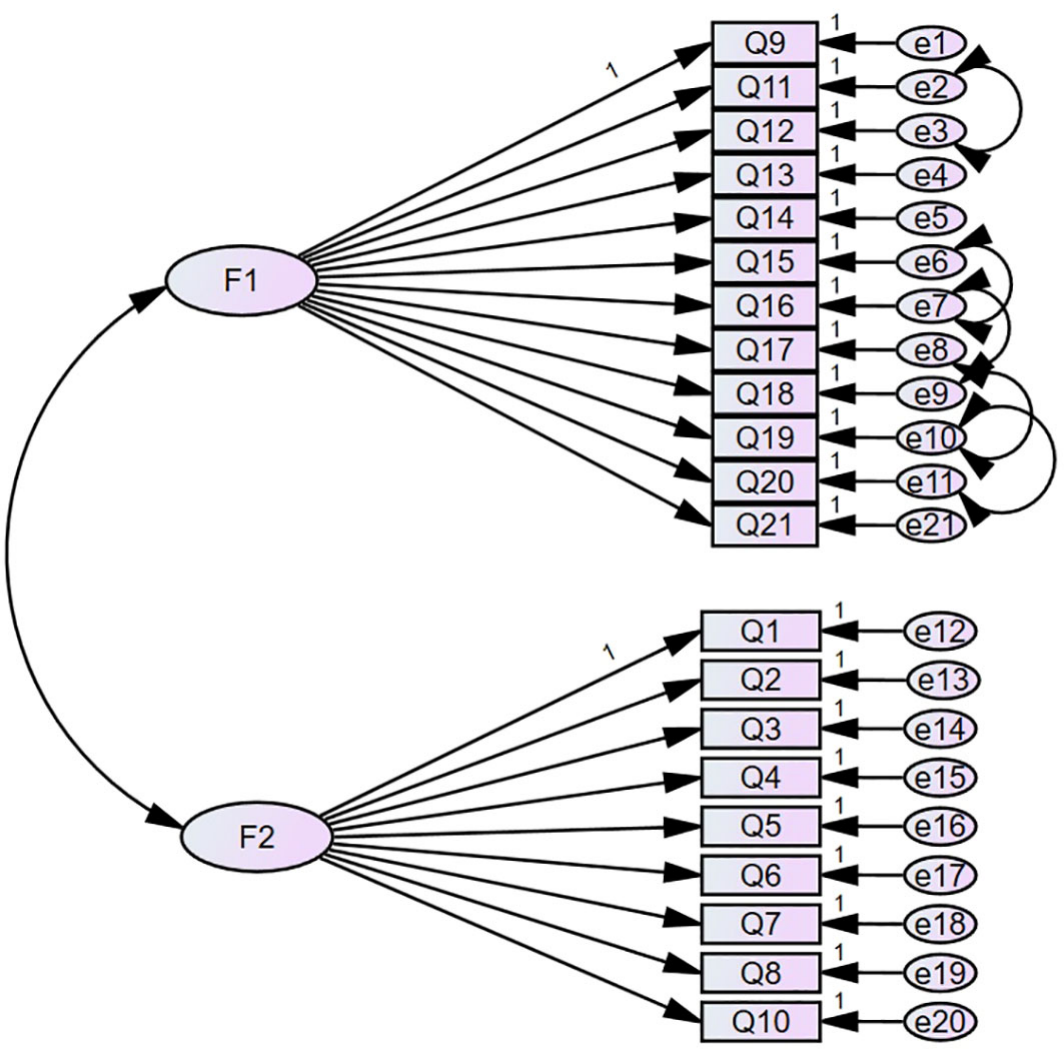
Confirmatory factor analysis (CFA) model of the EFS-HP.

Detailed results are presented in **Figure 1**; **Tables 4**–**6**.

**Table 4:** Results of the KMO measure and Bartlett’s test of sphericity.

KMO measure of sampling adequacy		0.969
Bartlett’s Test of Sphericity	χ²	7940.340
	Df	210
	P	0.001

**Table 5:** Exploratory factor analysis results.

Item	Factor 1	Factor 2
Q1	0.206	0.788
Q2	0.350	0.764
Q3	0.450	0.776
Q4	0.456	0.772
Q5	0.467	0.774
Q6	0.463	0.752
Q7	0.588	0.657
Q8	0.547	0.658
Q9	0.707	0.567
Q10	0.640	0.615
Q11	0.762	0.497
Q12	0.766	0.378
Q13	0.831	0.352
Q14	0.759	0.483
Q15	0.748	0.460
Q16	0.848	0.337
Q17	0.827	0.350
Q18	0.796	0.397
Q19	0.755	0.491
Q20	0.782	0.411
Q21	0.782	0.369

**Table 6:** Comparison of factor models.

Model	χ²/df	CFI	TLI	IFI	NFI	RMSEA
Single-factor model	5.355	0.844	0.827	0.845	0.816	0.153
Two-factor model	4.361	0.923	0.911	0.923	0.902	0.106

#### 3.4.3 Convergent validity

The total score of the Chinese EFS-HP was significantly and positively correlated with the CFS-MS total score (r = 0.587, P < 0.001), supporting convergent validity.

## 4 Discussion

This study cross-culturally adapted the Empathy Fatigue Scale for Health Professionals (EFS-HP) into Chinese and evaluated its psychometric properties in a sample of Chinese ICU nurses. The Chinese version retained all 21 original items and demonstrated good item discrimination, acceptable content validity, high internal consistency, and good split-half and test–retest reliability. Its total score correlated moderately and positively with that of the Compassion Fatigue Scale for Medical Staff (CFS-MS), supporting convergent validity. Taken together, these findings indicate that the Chinese EFS-HP is a promising instrument for assessing empathy fatigue among Chinese ICU nurses, although its factor structure requires further confirmation in larger, multicenter samples.

### 4.1 Content validity

Validity reflects the accuracy and relevance with which an instrument measures its intended construct and is typically appraised through both content and construct validity, the former indexing the relevance between items and their assigned dimensions. In this study, the overall content validity of the Chinese version was acceptable. Although the S-CVI/Ave fell below the recommended 0.90 threshold, it remained within a reasonable range (16). Nurses’ interpretation of certain items may have been shaped by occupational culture, linguistic conventions, and clinical experience; refining item wording to better reflect specific clinical scenarios could further strengthen content validity in future work (27, 28).e Chinese critical care context.

### 4.2 Construct validity

The Exploratory factor analysis (EFA) showed that the structure of the Chinese EFS-HP among Chinese ICU nurses diverged from the unidimensional structure of the original scale (19), a divergence likely shaped jointly by cultural background, occupational context, and patterns of nursing practice.

The comparison of CFA models indicated that the original single-factor model fit poorly, with CFI, TLI, IFI, and NFI all falling short of recommended standards. The two-factor model, by contrast, approached acceptable thresholds across these indices and yielded a relatively better fit, reflecting superior model adequacy. Although the RMSEA was higher than the ideal standard, it remained within the acceptable range and is therefore recommendable; cross-regional and multi-sample validation may further test its adaptability.

The EFA further suggested that the two extracted factors were relatively independent yet directed toward the same underlying construct. The first dimension captured the emotional intrusion and psychological pressure that ICU nurses experience during sustained empathic engagement—including emotional breakdown, difficulty in emotion regulation, blurred emotional boundaries, irritability and tension, negative affect, and an inability to disengage from patients’ distressing experiences (29)—and was accordingly labeled “Emotional Load.” The second dimension captured the depletion of physical and psychological resources arising from prolonged emotional investment, manifesting as physical fatigue, mental exhaustion, and diminished psychological energy after sustained exposure to patients’ emotional burdens (30); it was therefore labeled “Exhaustion State.”.

This structural difference may reflect the complexity of empathy fatigue within the Chinese ICU context. ICU nurses work continuously amid critically ill patients, high mortality risk, and demanding clinical decision-making, requiring them to sustain both emotional attentiveness and professional emotional regulation. Their experience of empathy fatigue may thus extend beyond emotional exhaustion to encompass continuous emotional involvement, difficulty maintaining emotional boundaries, and psychological intrusion (8, 10, 31).

Structural and cultural factors specific to the Chinese ICU setting may further reinforce this differentiation. On the one hand, restrictive visitation policies position nurses as partial emotional surrogates between patients and families, increasing their emotional labor (32). On the other hand, Chinese nursing culture emphasizes responsibility, professional dedication, and emotional restraint, compelling nurses to sustain professional expression under high-pressure conditions and potentially intensifying both emotional load and physical–psychological exhaustion (33, 34). The two-dimensional structure derived here may therefore more faithfully represent the psychological characteristics of empathy fatigue among Chinese ICU nurses.

Relative to the original unidimensional structure, this two-dimensional solution may better capture the complex psychological features of empathy fatigue in Chinese ICU nurses and may help clinical managers identify risk and deliver targeted interventions along the distinct dimensions of “Emotional Load” and “Exhaustion State.”.

### 4.3 Convergent validity

The Chinese EFS-HP correlated moderately and positively with the CFS-MS, indicating that both instruments tap prolonged emotional exposure and exhaustion. This association supports the specificity of the Chinese EFS-HP for assessing empathy fatigue among ICU nurses.

### 4.4 Reliability

Reliability denotes the consistency and stability of measurement results across time and contexts and is a key indicator of scale quality (35). The Chinese EFS-HP showed high internal consistency (Cronbach’s α=0.981), indicating strong item homogeneity and a stable representation of the same latent construct. Although an αα above 0.90 generally signifies excellent internal consistency, it may also point to potential item redundancy (36); given the inherently abstract nature of empathy fatigue, a degree of semantic proximity among items is to be expected. Split-half reliability further indicated good internal stability and high equivalence between the two halves of the scale (22), while test–retest reliability demonstrated good temporal stability. Collectively, these results confirm the favorable stability and consistency of the instrument’s measurements (37, 38).

## 5 Conclusion and limitations

### 5.1 Conclusion

The Chinese version of the EFS-HP comprises 21 items organized into two dimensions—Emotional Load and Exhaustion State. The instrument demonstrated good reliability and validity among Chinese ICU nurses and represents a practical, psychometrically sound tool for assessing empathy fatigue in critical care settings.

### 5.2 Limitations

This study has several limitations. First, the sample was drawn from a single tertiary hospital in Inner Mongolia using convenience sampling, and no external validation was performed, which may limit the generalizability of the findings. Future multicenter studies with more diverse populations are warranted to further confirm the stability of the factor structure.

Second, although confirmatory factor analysis (CFA) indicated acceptable incremental fit indices, the RMSEA value (0.106) exceeded the commonly recommended threshold of 0.08, suggesting suboptimal absolute model fit. Nevertheless, when interpreted in conjunction with other fit indices, the overall model fit was considered acceptable in the context of scale development; however, further refinement of item wording and model structure may improve model parsimony and fit.

Third, exploratory factor analysis (EFA) and confirmatory factor analysis (CFA) were conducted using the same sample rather than independent or randomly split subsamples. This may increase the risk of capitalizing on sample-specific characteristics and lead to overly optimistic estimates of model fit. Although this approach is sometimes used in scale development studies with limited sample sizes, future research should employ independent samples or cross-validation techniques to enhance the robustness and replicability of the factor structure.

Fourth, some items may have exhibited potential cross-loadings during factor extraction, which could indicate overlap between latent constructs and may affect the discriminant validity of the scale. Although efforts were made to retain the most theoretically and statistically appropriate item structure, further refinement of item allocation is recommended in future studies.

Finally, the cross-sectional design precludes examination of the longitudinal stability and responsiveness of the scale. In addition, measurement invariance across different subgroups (e.g., gender, professional titles, or clinical settings) was not assessed, and known-groups validity and cutoff scores were not established, which further limits the interpretability and practical application of the scale in clinical settings.

## Data Availability

The original contributions presented in the study are included in the article/**Supplementary Material**. Further inquiries can be directed to the corresponding author/s.
