# Supplementary material for: Cross-cultural adaptation and psychometric validation of the Chinese version of the empathy fatigue scale for health professionals among intensive care unit nurses
Source: Front Psychiatry. 2026 Jul 29;17:1918940. doi: 10.3389/fpsyt.2026.1918940 (PMC13462367; doi:10.3389/fpsyt.2026.1918940)
Supplement: Supplementary file 1 [file Table1.docx]

The Chinese Version of the EFS-HP Scale (5-point Likert format).

| 维度 | 条目 |
| --- | --- |
| 维度一 | 1.病人所经历的痛苦对我来说就像一个沉重的负担 |
|  | 2.同理心让我情绪崩溃 |
|  | 3. 当听到病人诉说痛苦经历时，我很难控制自己的情绪 |
|  | 4.因为我总是同情别人，所以很难设定自己的情感界限 |
|  | 5.试图理解病人的痛苦有时让我感到无助 |
|  | 6.当我同情病人时，我觉得这对我有负面影响 |
|  | 7.当我接触到病人的感情负担时，我会感到烦躁或紧张 |
|  | 8.病人所经历的困难深深地影响着我，我无法将他们从我的脑海中抹去 |
|  | 9.不断试图理解病人的感受是令人筋疲力尽的 |
|  | 10.当我过于关注病人的感受时，我感到情绪上筋疲力尽 |
|  | 11.对患者表现出同理心，有时对我来说感觉像是一项沉重的责任 |
|  | 12.我没有足够的时间来了解病人，因为我正试图了解病人的感受 |
| 维度二 | 1.当我过于关注患者的痛苦时，我会感到疲惫不堪 |
|  | 2.因为我不断地同情病人所经历的困难处境，我有时感到迷失了 |
|  | 3.在承担病人的感情负担时，我意识到我忽视了自己的感情 |
|  | 4.在试图支持病人时，我感到身体疲惫 |
|  | 5.当我不得不不断地同情别人的时候，我就会感到精神上的疲惫 |
|  | 6.当我过分关注病人的问题时，我认为我忽视了自己的需求 |
|  | 7.试图理解病人的感受有时对我来说是一个巨大的情感负担 |
|  | 8.当我对患者产生共情时，有时会感到无助和力不从心 |
|  | 9.我有时认为我忽视了自己的心理健康，因为我同情病人 |

Response scale: A 5-point Likert scale was used (1 = Strongly disagree, 2 = Disagree, 3 = Neither agree nor disagree, 4 = Agree, 5 = Strongly agree).
